# Monitoring vancomycin blood concentrations reduces mortality risk in critically ill patients: a retrospective cohort study using the MIMIC-IV database

**DOI:** 10.3389/fphar.2024.1458600

**Published:** 2024-11-14

**Authors:** Huaidong Peng, Yuantong Ou, Ruichang Zhang, Ruolun Wang, Deliang Wen, Qilin Yang, Xiaorui Liu

**Affiliations:** ^1^ Department of Pharmacy, The Second Affiliated Hospital, Guangzhou Medical University, Guangzhou, China; ^2^ Department of Critical Care, The Second Affiliated Hospital, Guangzhou Medical University, Guangzhou, China; ^3^ Department of Critical Care, Guangzhou Twelfth People’ Hospital, Guangzhou, China; ^4^ Department of Pharmacy, Guangzhou Institute of Cancer Research, The Affiliated Cancer Hospital, Guangzhou Medical University, Guangzhou, China

**Keywords:** vancomycin, therapeutic drug monitoring, critically ill patients, mortality, mimic iv

## Abstract

**Background:**

The incidence and mortality of severe Gram-positive cocci infections are particularly high in intensive care units (ICUs). Vancomycin remains the treatment of choice for severe infections caused by Gram-positive cocci, particularly methicillin-resistant *Staphylococcus aureus* (MRSA). Some guidelines recommend therapeutic drug monitoring (TDM) for critically ill patients treated with vancomycin; however, there is currently a lack of evidence to support that TDM improves the mortality rates of these patients. Therefore, we designed this cohort study to compare the impact of monitoring vancomycin blood concentrations on mortality rates in critically ill patients and to provide evidence to support this routine clinical practice.

**Methods:**

Data were extracted from the Medical Information Mart for Intensive Care (MIMIC)-IV database for a retrospective cohort analysis of critically ill patients receiving intravenous vancomycin treatment. The primary outcome was the 28 day mortality rate. The propensity score matching (PSM) method was used to match the baseline characteristics between patients in the TDM group and the non-TDM group. The relationship between 28 day mortality and vancomycin TDM in the critically ill cohort was evaluated using Cox proportional hazards regression analysis and Kaplan-Meier survival curves. Validation of the primary outcomes was conducted by comparing the PSM model and the Cox proportional hazards regression model. The robustness of the conclusion was subsequently verified by subgroup and sensitivity analyses.

**Results:**

Data for 18,056 critically ill patients who met the study criteria were collected from the MIMIC-IV database. Of these, 7,451 patients had at least one record of vancomycin blood concentration monitoring, which we defined as the TDM group. The TDM group exhibited a 28 day mortality rate of 25.7% (1,912/7,451) compared to 16.2% in the non-TDM group (1,723/10,605). After PSM, 4,264 patients were included in each of the TDM and non-TDM groups, with a 28 day mortality rate of 20.0% (1,022/4,264) in the TDM group and 26.4% (1,126/4,264) in the non-TDM group. Multivariate Cox proportional hazards analysis revealed a significantly lower 28 day mortality risk in the TDM group when compared to the non-TDM group (adjusted hazard ratio [HR]: 0.86; 95% confidence interval [CI]: 0.79, 0.93; *p* < 0.001). Further PSM analyses (adjusted HR: 0.91; 95% CI: 0.84, 0.99; *p* = 0.033) confirmed the lower risk of mortality in the TDM group. Kaplan-Meier survival analysis revealed a significantly higher survival rate at 28 days for the TDM group (log-rank test, *p* < 0.001). Subgroup analysis results indicated that patients with sepsis, septic shock, estimated glomerular filtration rate ≤ 60 mL/min/1.73 m^2^, undergoing renal replacement therapy, using vasoactive drugs, on mechanical ventilation, and those with higher severity scores (Acute Physiology Score III ≥40, Oxford Acute Severity of Illness Score ≥30, Simplified Acute Physiology Score II ≥ 30) significantly benefited from monitoring vancomycin blood concentrations. The results remained unchanged excluding patients staying in ICU for less than 48 h or those infected with MRSA.

**Conclusion:**

This cohort study showed that monitoring vancomycin blood concentrations is associated with a significantly lower 28 day mortality rate in critically ill patients, highlighting the importance of routinely performing vancomycin TDM in these patients.

## 1 Introduction

Severe infections caused by Gram-positive cocci are prevalent in intensive care units (ICUs), with reported incidence rates ranging from 16.1% to 50% (Vincent et al., 2009; Baykara et al., 2018; Martin et al., 2003; Chen et al., 2023; Tang et al., 2023). In particular, Gram-positive cocci have been isolated from respiratory specimens in more than 50% of cases with ventilator-associated pneumonia (Yoshimura et al., 2022). These infections are often accompanied by high mortality (Hanberger et al., 2011; van Hal et al., 2012). For instance, bacteremia caused by methicillin-resistant *Staphylococcus aureus* (MRSA) has a 30 day mortality rate of 28%–30.5% (Wang et al., 2010; Yaw et al., 2014). Vancomycin, a glycopeptide antibiotic, has demonstrated efficacy against severe infections caused by Gram-positive cocci, including MRSA (Jeffres, 2017). Furthermore, it has been the treatment of choice for MRSA for decades and remains one of the most frequently used antibiotics in ICUs (Magill et al., 2014). However, due to a narrow therapeutic window, significant inter-individual pharmacokinetic (PK) variability, and dose-dependent nephrotoxicity, some guidelines recommend therapeutic drug monitoring (TDM) for critically ill patients or those with severe MRSA infections treated with vancomycin (Ye et al., 2016; He et al., 2020; Reuter et al., 2022; Abdul-Aziz et al., 2020; Rybak et al., 2020).

In a narrow sense, vancomycin TDM focuses on adjusting dosage based on blood drug concentration measurements, primarily trough levels, to ensure effective treatment while minimizing risk of toxicity. Trough levels, the lowest drug concentration before the next dose, are recommended by the 2009 clinical guideline to be maintained between 10 and 20 mg/L to balance efficacy and toxicity (Rybak et al., 2009). More broadly, vancomycin TDM includes both trough concentration monitoring and the area under the concentration-time curve (AUC), which offers a more comprehensive assessment of drug exposure and correlates better with both efficacy and toxicity. As a result, AUC monitoring has been increasingly incorporated into TDM standards, enabling more precise dose adjustments and individualized treatment strategies, as seen in the 2020 guideline (Rybak et al., 2020). Trough concentration monitoring offers the advantages of simplicity and quick results, as it involves direct measurement. In contrast, traditional AUC monitoring requires multiple blood samples at different time points, followed by nonlinear curve fitting, making it impractical in clinical settings due to the need for repeated sampling (Yamada et al., 2023). Currently, clinical practice primarily uses the peak-trough two-point method and Bayesian software for AUC estimation. The two-point method involves collecting peak and trough concentrations within a dosing interval, followed by calculating AUC using a first-order equation (Meng et al., 2019). The Bayesian method uses a population PK model combined with 1-2 blood concentration measurements to estimate AUC(Rybak et al., 2020; Turner et al., 2018). Thus, regardless of the method, AUC monitoring requires at least 1-2 blood concentration measurements, making blood concentration monitoring essential for accurate AUC estimation. Therefore, as long as blood concentration measurements are available, it is possible to determine if a patient underwent vancomycin TDM, whether through trough level or AUC monitoring.

Emerging evidence supports a specific relationship between nephrotoxicity and both trough concentration (van Hal et al., 2013; Bellos et al., 2020) and the AUC to the minimum inhibitory concentration (AUC/MIC) (Aljefri et al., 2019; Abdelmessih et al., 2022) during the treatment of serious infections caused by *Staphylococcus aureus*, particularly MRSA. Although vancomycin TDM can effectively reduce the incidence of nephrotoxicity, trough concentrations do not appear to predict clinical efficacy accurately (Lodise et al., 2008; Steinmetz et al., 2015; Prybylski, 2015). A previous systematic review, incorporating data from over 2,000 patients with invasive MRSA infections, found no significant difference in all-cause mortality between patient groups with trough concentrations >10 mg/L compared to those with levels ≥15 mg/L (Steinmetz et al., 2015). Similarly, a previous meta-analysis that included more than 1,600 patients with *Staphylococcus aureus* bacteremia demonstrated that trough concentrations >15 mg/L did not correlate with reduced rates of treatment failure, duration of bacteremia, or mortality (Prybylski, 2015). It is generally believed that the AUC/MIC ratio is superior to trough concentration in predicting survival outcomes and clinical cure, while this ratio only shows moderate levels of sensitivity and specificity (Lodise et al., 2008; Dalton et al., 2020; Tsutsuura et al., 2021). The multicenter prospective PROVIDE study further elucidated the relationship between initial vancomycin exposure and clinical treatment failure rates in adult patients with MRSA bacteremia, highlighting that higher AUC/MIC ratios do not necessarily translate into a reduced risk of treatment failure but are linked to an increased incidence of acute kidney injury (Lodise et al., 2020). There is still a clear lack of robust data correlating AUC with mortality; furthermore, the available evidence on the impact of vancomycin TDM on mortality rates is highly limited (Flannery et al., 2021; Briassoulis and Briassoulis, 2022). This highlights the necessity for further rigorous studies to clarify these relationships and optimize vancomycin TDM strategies in clinical settings.

In ICUs, vancomycin is predominantly administered empirically by clinicians based on the severity of infection, the prevalence of local pathogens, and patterns of antibiotic resistance without results generated by pathogen culture (Jones et al., 2020; Cowley et al., 2019). This practice has generated clinical debate about whether routine TDM of vancomycin is necessary for all critically ill patients (Darko et al., 2003). In particular, we need to know whether routine TDM can reduce the mortality rate of these patients. Although some guidelines advocate the use of TDM during vancomycin therapy in critical settings, these recommendations are based on limited clinical and safety data and do not consider the costs associated with vancomycin TDM. Currently, there is insufficient evidence to evaluate the impact of this practice on mortality and cost-effectiveness (Ye et al., 2016; He et al., 2020; Reuter et al., 2022; Abdul-Aziz et al., 2020; Rybak et al., 2009). In the updated 2020 vancomycin TDM guideline, the Therapeutic Drug Monitoring Committee of the Chinese Pharmacological Society reported that despite recommendations for TDM in critically ill patients, these were classified as low-quality evidence due to the lack of supportive data (He et al., 2020). Some previous studies reported that routine vancomycin TDM may consume significant time and healthcare resources, and may potentially lack cost-effectiveness (Jeffres, 2017; Darko et al., 2003). Consequently, in the present study, we utilized a large dataset from the MIMIC-IV database to investigate the impact of monitoring vancomycin blood concentrations on mortality rates in critically ill patients. Our hypothesis was that monitoring vancomycin blood concentrations would reduce mortality. If our hypothesis is proven, our findings would provide the most direct evidence to support the routine implementation of vancomycin TDM in critically ill patients.

## 2 Materials and methods

### 2.1 Data source

The Medical Information Mart for Intensive Care IV (MIMIC-IV) database, hosted at PhysioNet, is a publicly accessible, single-center repository that includes data relating to 730,141 ICU admissions at the Beth Israel Deaconess Medical Center in the United States, spanning from 2008 to 2019 (Johnson et al., 2023). Researcher Huaidong Peng (certification number: 59679596) and colleagues utilized the database to extract a wide range of clinical data, including patient demographics, vital signs, laboratory tests, comorbidities, severity of illness scores, therapeutic interventions, and specifics regarding vancomycin administration and TDM information. The use of the MIMIC-IV database was authorized by the Institutional Review Boards of the Massachusetts Institute of Technology and Beth Israel Deaconess Medical Center. Our study was conducted in accordance with the Declaration of Helsinki, and due to the anonymization of participant data and its standardized format, additional approval from the ethics committee was deemed unnecessary.

### 2.2 Study population

**FIGURE 1:**
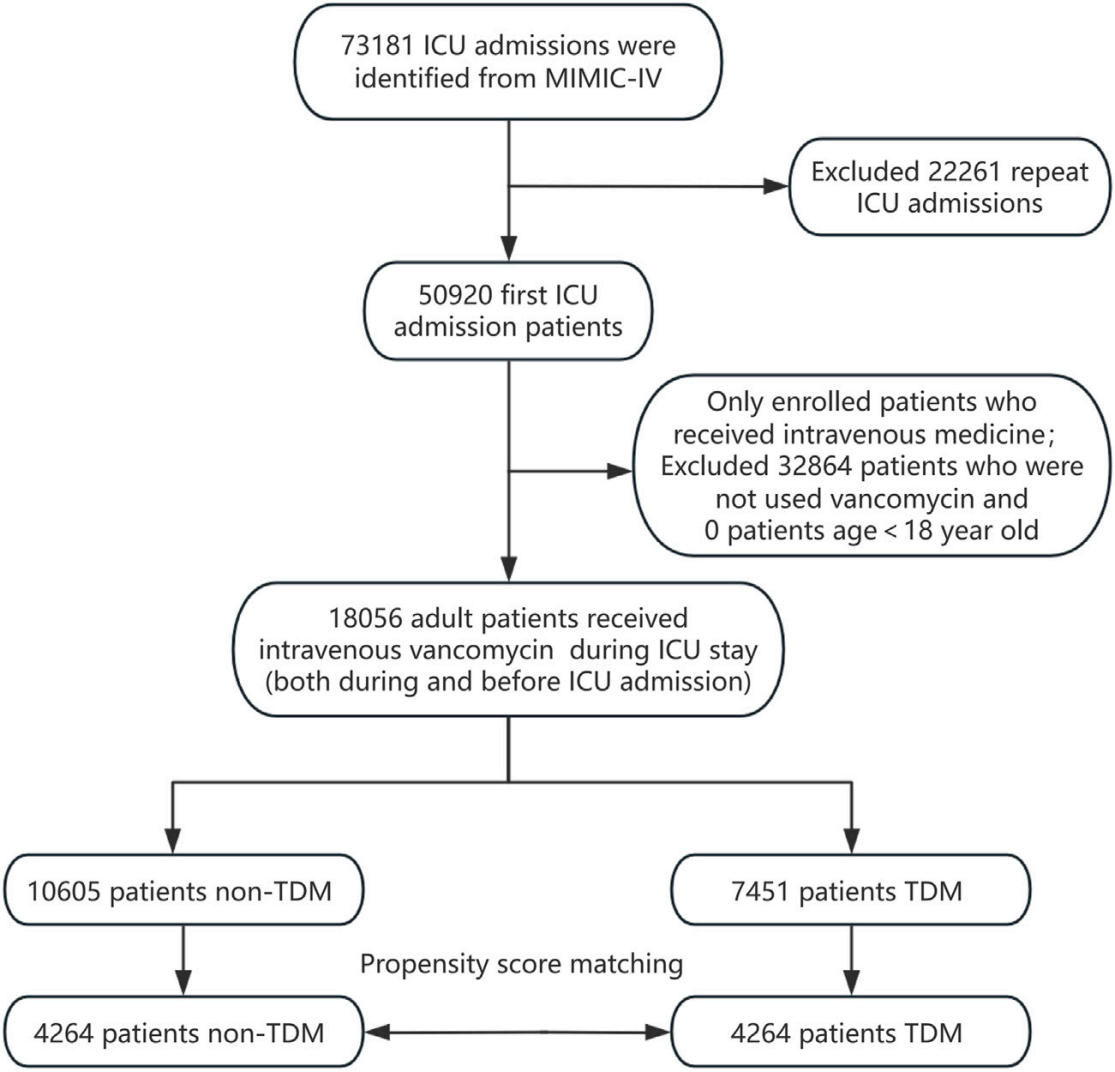
Flow chart depicting the enrollment process for patients.

All patients recorded in the MIMIC-IV database were considered eligible for inclusion in this study. For those with multiple ICU admissions, only the data from their first ICU stay were analyzed. We included patients who began intravenous vancomycin treatment either before or after ICU admission. Vancomycin TDM was defined as having at least one blood vancomycin concentration measurement during the ICU stay, irrespective of whether it was a trough, peak, or random concentration. Patients receiving vancomycin TDM during their ICU hospitalization were designated as the experimental group (the TDM group), while those administered vancomycin without subsequent TDM formed the control group (the non-TDM group). Additionally, the analysis was limited to adult patients aged 18 years and older. The patient enrollment process for this study is depicted in Figure 1.

### 2.3 Covariates and outcome

We used Structured Query Language to systematically extract patient data from the database. The extracted data included a range of variables: basic demographic details for hospital admission registration such as gender, age, and race; vital signs, including heart rate, mean arterial pressure, respiratory rate, temperature, and SpO2; laboratory tests, including white blood cell count (WBC), hemoglobin, hematocrit, platelets, creatinine, blood urea nitrogen (BUN), finger glucose, potassium, and bicarbonate; comorbidities, including hypertension, congestive heart failure, chronic obstructive pulmonary disease (COPD), liver disease, diabetes, renal disease, and malignant cancer; severity of illness scores, including the Acute Physiology Score (APS) III, Simplified Acute Physiology Score (SAPS) II, Sequential Organ Failure Assessment (SOFA) score, Charlson Comorbidity Index, and Oxford Acute Severity of Illness Score (OASIS); therapeutic interventions such as renal replacement therapy (RRT), vasoactive drugs, and mechanical ventilation; and vancomycin administration and TDM information. The estimated glomerular filtration rate (eGFR) was calculated using the Chronic Kidney Disease Epidemiology Collaboration (CKD-EPI) formula, which adjusts for serum creatinine while accounting for the patient’s gender, age, and creatinine levels (Inker et al., 2021).

The primary outcome of this study was 28 day mortality. Secondary outcomes included ICU mortality, hospital mortality, 60 day mortality, and 90 day mortality.

### 2.4 Statistical analysis

We addressed missing data using K-Nearest Neighbors imputation (Faisal and Tutz, 2022), detailed information relating to missing data is provided in Supplementary Table 1. Continuous variables are reported as either mean ± standard deviation or median with interquartile range (IQR), while categorical variables are reported as frequency (percentage). The conformity of continuous variables to the normal distribution was tested using the Shapiro-Wilk test or Kolmogorov-Smirnov test. For continuous variables that conformed to a normal distribution, we used the Student’s t-test to compare means. For non-normally distributed continuous variables, the Wilcoxon’s rank-sum test was applied. For categorical variables, we used Pearson’s chi-squared test or Fisher’s exact test, as appropriate.

Cox proportional hazards regression analyses were used to investigate the independent association between vancomycin TDM and 28 day mortality, yielding hazard ratios (HRs) and 95% confidence intervals (CIs). Survival distributions were estimated by Kaplan-Meier analysis, and differences were assessed by the log-rank test. To balance baseline characteristics between the TDM and non-TDM groups, we implemented propensity score matching (PSM) using a 1:1 nearest neighbor matching algorithm with a caliper width of 0.1. The variables listed in Table 1 were used to generate the propensity score. The primary outcome was further validated using the PSM model, which applied the estimated propensity scores as weights. The results were then compared to those derived from the Cox proportional hazards regression model.

**TABLE 1:** Baseline characteristics of the patients enrolled from the MIMIC-IV database.

Patient characteristic	Before PSM				After PSM			
	Total (n = 18,056)	Non-TDM group (n = 10,605)	TDM group (n = 7,451)	SMD	Total (n = 8,528)	Non-TDM group (n = 4,264)	TDM group (n = 4,264)	SMD
Gender [male, n (%)]	10,599 (58.7)	6,220 (58.7)	4,379 (58.8)	0.002	4,844 (56.8)	2,422 (56.8)	2,422 (56.8)	<0.001
Age (years)	66.0 ± 16.0	67.2 ± 15.5	64.4 ± 16.6	0.178	66.1 ± 16.4	66.2 ± 16.4	66.1 ± 16.5	0.006
RACE [white, n (%)]	11,939 (66.1)	7,251 (68.4)	4,688 (62.9)	0.115	5,509 (64.6)	2,737 (64.2)	2,772 (65)	0.017
Vital signs								
Heart rate (bpm)	87.4 ± 16.1	85.8 ± 15.0	89.7 ± 17.2	0.242	88.5 ± 16.6	88.7 ± 16.5	88.2 ± 16.7	0.03
MAP (mmHg)	76.3 ± 10.1	76.0 ± 9.8	76.7 ± 10.5	0.064	76.5 ± 10.6	76.5 ± 10.7	76.5 ± 10.4	0.004
Respiratory rate (/min)	19.9 ± 4.1	19.4 ± 3.9	20.7 ± 4.3	0.329	20.3 ± 4.2	20.4 ± 4.3	20.3 ± 4.1	0.019
Temperature (°C)	37.5 ± 0.8	37.4 ± 0.8	37.7 ± 0.9	0.294	37.6 ± 0.9	37.6 ± 0.9	37.6 ± 0.9	0.008
Spo2(%)	96.9 ± 2.5	96.9 ± 2.6	96.8 ± 2.4	0.049	96.8 ± 2.6	96.7 ± 2.9	96.8 ± 2.4	0.028
Laboratory tests								
WBC(×10^9^)	14.4 (10.3, 19.6)	14.3 (10.3, 19.0)	14.8 (10.5, 20.3)	0.081	14.4 (10.2, 19.8)	14.5 (10.1, 19.7)	14.4 (10.3, 19.9)	0.011
Hemoglobin (g/L)	9.8 ± 2.1	9.8 ± 2.1	9.8 ± 2.2	0.022	9.8 ± 2.2	9.8 ± 2.2	9.8 ± 2.2	0.007
Hematocrit (%)	29.7 ± 6.3	29.5 ± 6.1	29.9 ± 6.6	0.067	29.8 ± 6.5	29.9 ± 6.6	29.8 ± 6.5	0.015
Platelets (×10^9^)	161.0 (111.0, 228.0)	157.0 (113.0, 220.2)	169.0 (108.0, 237.5)	0.074	169.0 (112.0, 238.0)	168.0 (112.0, 236.0)	170.0 (112.0, 240.0)	0.004
eGFR (mL/min/1.73 m^2^)	61.2 (34.4, 88.0)	67.9 (42.9, 89.9)	50.7 (24.6, 83.6)	0.347	51.8 (27.7, 82.1)	52.9 (29.7, 82.7)	50.8 (25.1, 81.4)	0.075
BUN(mg/dL)	22.0 (15.0, 36.0)	19.0 (14.0, 30.0)	26.0 (17.0, 45.0)	0.398	25.0 (16.0, 41.0)	25.0 (16.0, 41.0)	24.0 (16.0, 41.0)	0.021
Glucose (finger,mg/dL)	132.0 (115.5, 159.5)	130.4 (116.4, 152.8)	135.9 (114.0, 170.0)	0.047	134.0 (113.6, 167.2)	135.2 (115.0, 169.0)	133.0 (112.3, 165.5)	0.021
Potassium (mmol/L)	3.9 ± 0.6	3.9 ± 0.6	3.9 ± 0.6	0.108	3.9 ± 0.6	3.9 ± 0.6	3.9 ± 0.6	0.007
Bicarbonate (mmol/L)	21.0 ± 4.9	21.4 ± 4.5	20.3 ± 5.3	0.222	20.6 ± 5.2	20.6 ± 5.4	20.7 ± 5.1	0.007
Comorbidity diseases, n(%)								
Hypertension	11,448 (63.4)	6,922 (65.3)	4,526 (60.7)	0.094	5,269 (61.8)	2,623 (61.5)	2,646 (62.1)	0.011
Congestive heart failure	5,250 (29.1)	2,933 (27.7)	2,317 (31.1)	0.076	2,675 (31.4)	1,344 (31.5)	1,331 (31.2)	0.007
COPD	4,654 (25.8)	2,619 (24.7)	2035 (27.3)	0.06	2,322 (27.2)	1,185 (27.8)	1,137 (26.7)	0.025
Liver disease	2,554 (14.1)	1,134 (10.7)	1,420 (19.1)	0.237	1,374 (16.1)	704 (16.5)	670 (15.7)	0.022
Diabetes	4,428 (24.5)	2,623 (24.7)	1805 (24.2)	0.012	2068 (24.2)	1,034 (24.2)	1,034 (24.2)	<0.001
Renal disease	3,707 (20.5)	1875 (17.7)	1832 (24.6)	0.17	2058 (24.1)	1,044 (24.5)	1,014 (23.8)	0.016
Malignant cancer	2,526 (14.0)	1,427 (13.5)	1,099 (14.7)	0.037	1,373 (16.1)	699 (16.4)	674 (15.8)	0.016
Sepsis	14,053 (77.8)	7,227 (68.1)	6,826 (91.6)	0.612	7,481 (87.7)	3,780 (88.6)	3,701 (86.8)	0.056
Septic shock	8,236 (45.6)	3,786 (35.7)	4,450 (59.7)	0.496	4,212 (49.4)	2,137 (50.1)	2075 (48.7)	0.029
Severity of illness scores								
CCI	6.0 (4.0, 8.0)	5.0 (4.0, 7.0)	6.0 (4.0, 8.0)	0.09	6.0 (4.0, 8.0)	6.0 (4.0, 8.0)	6.0 (4.0, 8.0)	0.022
SOFA score	5.0 (3.0, 8.0)	5.0 (3.0, 7.0)	6.0 (4.0, 9.0)	0.454	6.0 (3.0, 8.0)	5.0 (3.0, 8.0)	6.0 (3.0, 8.0)	0.034
APS III	50.0 (35.0, 71.0)	42.0 (31.0, 59.0)	63.0 (46.0, 84.0)	0.737	54.0 (40.0, 73.0)	54.0 (40.0, 75.0)	55.0 (41.0, 72.0)	0.049
SAPS II	39.9 ± 15.0	37.9 ± 14.3	42.8 ± 15.4	0.335	41.4 ± 15.4	41.7 ± 16.0	41.1 ± 14.9	0.04
OASIS	34.9 ± 9.6	32.6 ± 8.9	38.3 ± 9.5	0.619	36.0 ± 9.1	36.2 ± 9.2	35.8 ± 9.1	0.042
Therapy, n(%)								
RRT	928 (5.1)	260 (2.5)	668 (9)	0.284	470 (5.5)	229 (5.4)	241 (5.7)	0.012
Mechanical ventilation	10,423 (57.7)	5,174 (48.8)	5,249 (70.4)	0.453	4,981 (58.4)	2,492 (58.4)	2,489 (58.4)	0.001
Vasoactive drug	10,034 (55.6)	5,407 (51)	4,627 (62.1)	0.226	4,484 (52.6)	2,248 (52.7)	2,236 (52.4)	0.006
Infectious pathogen, n (%)								
MRSA	1,307 (7.2)	541 (5.1)	766 (10.3)	0.195	672 (7.9)	329 (7.7)	343 (8)	0.012
Details of first administration of vancomycin in ICU								
Accumulated dose(g)	2.0 (1.25, 4.0)	2.0 (1.0, 3.0)	4.0 (2.0, 6.0)	0.843	2.75 (1.875, 4.0)	2.0 (1.5, 4.0)	3.0 (2.0, 4.125)	0.071
Medication time(d)	2.7 (1.7, 6.0)	2.0 (1.2, 3.1)	5.2 (2.7, 10.0)	0.624	3.0 (1.7, 6.1)	2.5 (1.2, 5.3)	3.5 (2.1, 6.6)	0.087

MAP, mean arterial pressure; Spo2, percutaneous arterial oxygen saturation; WBC, white blood cell count; eGFR, estimated glomerular filtration rate; BUN, blood urea nitrogen; COPD, chronic obstructive pulmonary disease; CCI, charlson comorbidity score; SOFA, score, sequential organ failure score; APS III, acute physiology score III; SAPS II, simplified acute physiology score II; OASIS, oxford acute severity of illness score; RRT, renal replacement therapy; MRSA, methicillin-resistant *Staphylococcus aureus*.

All statistical analyses were conducted using R statistical software, version 3.3.2 (http://www.R-project.org, The R Foundation) and Free Statistics software, version 1.9 (https://www.clinicalscientists.cn/freestatistics/). A two-tailed test approach was adopted, and *p* < 0.05 was considered statistically significant.

### 2.5 Subgroup analysis and sensitivity analysis

To evaluate the robustness of our findings, we conducted subgroup analyses stratified by gender, age, race, sepsis, septic shock, eGFR, RRT, the use of vasoactive drugs, mechanical ventilation, APS III, OASIS, and SAPS II. Furthermore, to delineate independent associations, we performed distinct sensitivity analyses. Sensitivity analysis was performed after excluding patients with ICU hospitalization time less than 48 h or diagnosed with MRSA infection. These analyses aimed to ensure the reliability and applicability of our results across diverse patient groups and clinical scenarios.

## 3 Results

### 3.1 Patient characteristics

A total of 18,056 patients who received intravenous vancomycin were enrolled in our cohort from the MIMIC-IV database. The mean age of the patients was 66.0 ± 16.0 years, and 58.7% were male (n = 10,599). The overall 28 day mortality rate was 20.1% (3,635/18,056). Of these, 7,451 patients (41.3%) underwent at least one round of vancomycin blood concentration monitoring, while 10,605 (58.7%) patients did not (Table 1). Table 1 presents the clinical information of both the non-TDM and TDM groups before and after PSM. When considering all enrolled patients, those in the TDM group were older, had higher WBC counts, lower eGFR levels, higher BUN levels, and a higher proportion of comorbidities such as heart failure, COPD, liver disease, and kidney disease when compared to the non-TDM group (all *p* < 0.001). The incidence of sepsis and septic shock was higher in the TDM group, and all five severity scores were higher than those in the non-TDM group (all *p* < 0.001). Additionally, there was a higher proportion of patients with definitive MRSA infections, as well as more patients requiring RRT, mechanical ventilation, and vasoactive drug treatment (all *p* < 0.001). These data suggest that patients in the TDM group were in more severe condition than those in the non-TDM group.

After performing PSM, the characteristics of 4,264 patients were successfully matched between the two groups. The standardized mean differences (SMD) of all variables after PSM were less than 10%, indicating good quality of the matched samples (Table 1). After minimizing the interference of confounders through PSM, monitoring vancomycin blood concentrations was identified as the main factor.

### 3.2 Primary outcome

Table 2 presents the mortality data arising from our analysis. According to the MIMIC-IV database, the 28 day mortality rate for critically ill patients treated with vancomycin was 20.1% (3,635/18,056). Notably, the 28 day mortality rate in the TDM group was 25.7% (1,912/7,451), which was significantly higher than 16.2% (1,723/10,605) in the non-TDM group (*p* < 0.001). After PSM, the overall 28 day mortality rate was 25.2% (2,148/8,528). However, within the matched cohort, the 28 day mortality rate of the TDM group was significantly lower than that of the non-TDM group (20.0% vs 26.4%, *p* = 0.009).

**TABLE 2:** Primary outcome and secondary outcomes of the study.

Outcomes	Matching	Total	Non-TDM group	TDM group	*p*
28 day mortality	before PSM, n (%)	3,635/18,056 (20.1)	1723/10,605 (16.2)	1912/7,451 (25.7)	<0.001
	after PSM, n (%)	2,148/8,528 (25.2)	1,126/4,264 (26.4)	1,022/4,264 (20.0)	0.009
ICU mortality	before PSM, n (%)	2,309/18,056 (12.8)	1,053/10,605 (9.9)	1,256/7,451 (16.9)	<0.001
	after PSM, n (%)	1,355/8,528 (15.9)	757/4,264 (17.8)	598/4,264 (14.0)	<0.001
hospital mortality	before PSM, n (%)	3,088/18,056 (17.1)	1,401/10,605 (13.2)	1,687/7,451 (22.6)	<0.001
	after PSM, n (%)	1828/8,528 (21.4)	994/4,264 (23.3)	834/4,264 (19.6)	<0.001
60 day mortality	before PSM, n (%)	4,359/18,056 (24.1)	2066/10,605 (19.5)	2,293/7,451 (30.8)	<0.001
	after PSM, n (%)	2,544/8,528 (29.8)	1,324/4,264 (31.1)	1,220/4,264 (28.6)	0.014
90 day mortality	before PSM, n (%)	4,739/18,056 (26.2)	2,241/10,605 (21.1)	2,498/7,451 (33.5)	<0.001
	after PSM, n (%)	2,753/8,528 (32.3)	1,411/4,264 (33.1)	1,342/4,264 (31.5)	0.110

### 3.3 Secondary outcomes

ICU mortality, hospital mortality, 60 day mortality, and 90 day mortality, all showed patterns that were similar to the 28 day mortality results (Table 2). After PSM, all of these mortality metrics shifted from being higher in the TDM group than the non-TDM group before PSM to being significantly lower in the TDM group after PSM, with all differences were significant (*p* < 0.05).

### 3.4 Association between vancomycin TDM and 28 day mortality

In extended multivariable Cox regression models, we made adjustments using various covariates, and the changes in the HRs are detailed in Table 3. After incorporating all covariates, multivariate Cox proportional hazards regression analysis revealed an adjusted HR of 0.86 (95% CI: 0.79, 0.93; *p* < 0.001), indicating a significant reduction in hazard. This finding was consistent with the post-matched cohort results, where PSM (adjusted HR: 0.91; 95% CI: 0.84, 0.99; *p* = 0.033) showed that monitoring vancomycin blood concentrations was significantly associated with reduced 28 day mortality among critically ill patients (Table 4). This robust statistical evidence highlights the efficacy of vancomycin TDM in improving survival outcomes in this vulnerable patient population.

**TABLE 3:** Association between TDM and 28 day mortality using Cox proportional hazards regression analysis.

	Adjusted HR	95% CI	*p*-value
Model 1	1.59	(1.49 ∼ 1.70)	<0.001
Model 2	1.67	(1.56 ∼ 1.78)	<0.001
Model 3	1.18	(1.10 ∼ 1.27)	<0.001
Model 4	1.13	(1.05 ∼ 1.21)	0.001
Model 5	0.91	(0.84 ∼ 0.98)	<0.001
Model 6	0.84	(0.78 ∼ 0.90)	<0.001
Model 7	0.68	(0.63 ∼ 0.73)	<0.001
Model 8	0.86	(0.79 ∼ 0.93)	<0.001

Adjusted covariates: Model 1 = Vancomycin TDM, only; Model 2 = Model 1+(Gender, Age, Race); Model 3 = Model 2+(Heart rate, MAP, respiratory rate, Temperature, and SpO2)+(WBC, hemoglobin, Hematocrit, Platelets, Creatinine; BUN, finger glucose, Potassium, and Bicarbonate); Model 4 = Model 3+(Hypertension, Congestive heart failure, COPD, liver disease, Diabetes, Renal disease, and Malignant cancer); Model 5 = Model 4+(RRT, vasoactive drug, Mechanical ventilation, and MRSA); Model 6 = Model 5+(Sepsis, Septic shock); Model 7 = Model 6+(CCI, SOFA, score, APS III, SAPS II, OASIS); Model 8 = Model 7+(Accumulated dose, Medication time).

**TABLE 4:** The association between vancomycin TDM and 28 day mortality, as determined by analyses incorporating multiple models.

	HR	95% CI	*p*-value
Crude analysis.Unmatched	1.59	(1.49 ∼ 1.70)	<0.001
Multivariable.adjusted^a^	0.86	(0.79 ∼ 0.93)	<0.001
PropensityScore.Matched^b^	0.85	(0.78 ∼ 0.92)	<0.001
PropensityScore.adjusted^c^	0.91	(0.84 ∼ 0.99)	0.033

^a^ HR, from a multivariable Cox proportional model adjusted for all covariates in Table 1.

^b^ HR, from a multivariate Cox proportional hazards model with the same strata and covariates matched according to the propensity score.

^c^ HR, from a multivariable Cox proportional hazards model with the same strata and covariates, with additional adjustment for the propensity score.

**FIGURE 2:**
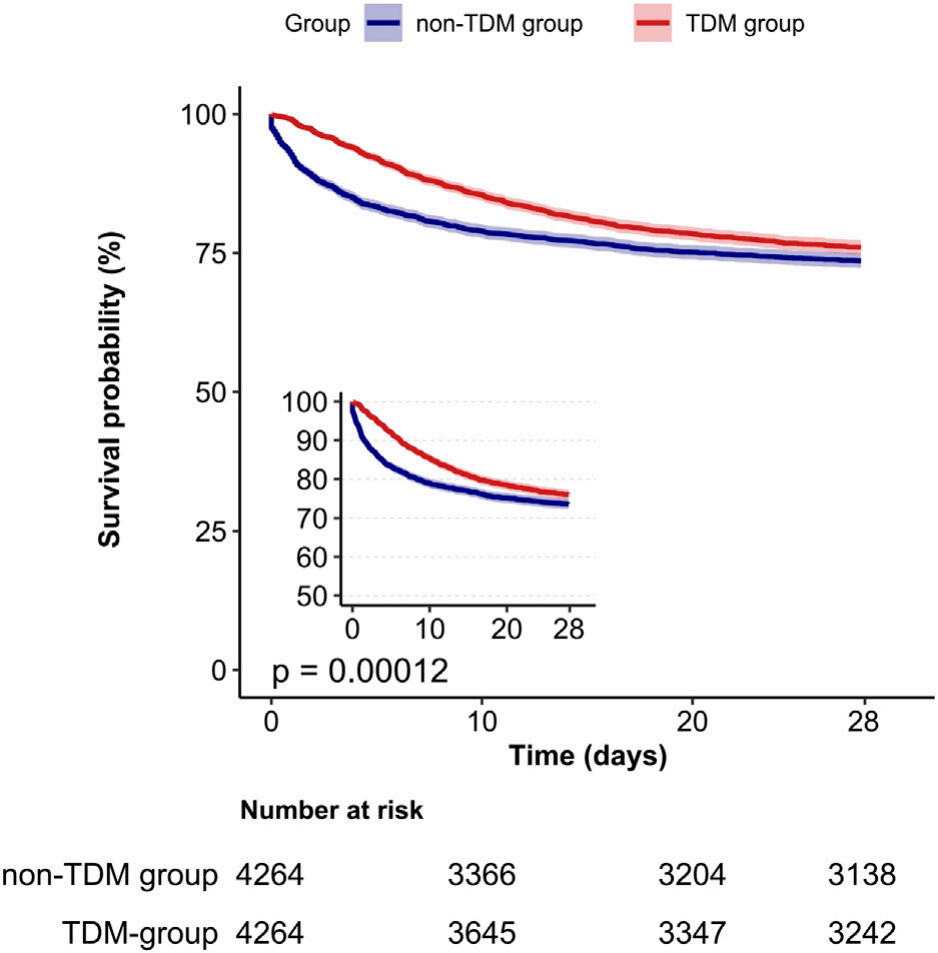
Kaplan-Meier survival curves for 28-day mortality in critically ill patients from the TDM group and the non-TDM group.

A Kaplan-Meier survival curve also demonstrated that the TDM group had a lower 28 day mortality rate (log-rank test: *p* < 0.001; Figure 2). Results from the 60 day and 90 day survival curves were consistent with those from the 28 day results (detailed results are available in Supplementary Images 1, 2).

### 3.5 Subgroup analysis

**FIGURE 3:**
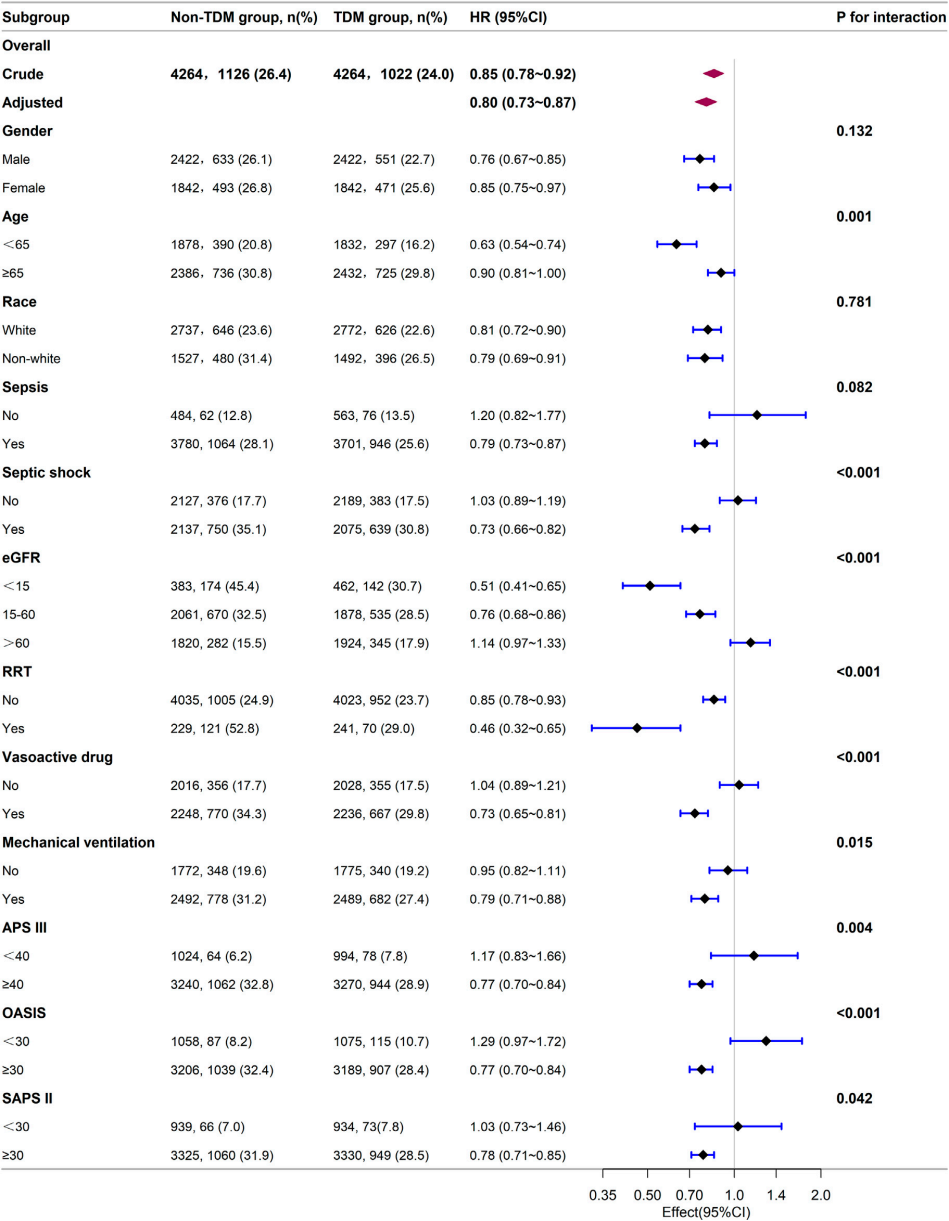
Subgroup analysis of the relationship between vancomycin TDM and 28-day mortality, as visualized by a forest plot.

Next, we stratified our cohort into various demographic and clinical subgroups based on gender, age, race, sepsis, septic shock, eGFR, RRT, the use of vasoactive drugs, mechanical ventilation, APS III, OASIS, and SAPS II. The impact of monitoring vancomycin blood concentrations on 28 day mortality was investigated and the results were visualized as a forest plot (Figure 3). Subgroup analyses indicated a general negative correlation between monitoring vancomycin blood concentrations and 28 day mortality among critically ill patients. The results showed lower 28 day mortality in several subgroups, including males (HR: 0.76; 95% CI: 0.67, 0.85), females (HR: 0.85; 95% CI: 0.75, 0.97), patients younger than 65 years (HR: 0.63; 95% CI: 0.54, 0.74), those aged 65 years or older (HR: 0.90; 95% CI: 0.81, 1.00), White individuals (HR: 0.81; 95% CI: 0.72, 0.90), non-White individuals (HR: 0.79; 95% CI: 0.69, 0.91), patients with sepsis (HR: 0.79; 95% CI: 0.73, 0.87), those with septic shock (HR: 0.73; 95% CI: 0.66, 0.82), patients with eGFR <15 mL/min/1.73 m^2^ (HR: 0.51; 95% CI: 0.41, 0.65), those with eGFR between 15 and 60 mL/min/1.73 m^2^ (HR: 0.76; 95% CI: 0.68, 0.86), patients undergoing RRT (HR: 0.46; 95% CI: 0.32, 0.65), those receiving vasoactive drugs (HR: 0.73; 95% CI: 0.65, 0.81), those on mechanical ventilation (HR: 0.79; 95% CI: 0.71, 0.88), and patients with APS III scores ≥40 (HR: 0.77; 95% CI: 0.70, 0.84), OASIS scores ≥30 (HR: 0.77; 95% CI: 0.70, 0.84), or SAPS II scores ≥30 (HR: 0.78; 95% CI: 0.71, 0.85). However, interactions were identified between age, septic shock, RRT, vasoactive drugs, vasoactive drugs, APS III, OASIS, and SAPS II (*p* for interaction <0.05) (Figure 3).

### 3.6 Sensitivity analysis

There were 18,056 patients in the entire cohort. After excluding 6,058 patients whose ICU stay was less than 48 h, 11,998 patients remained for final analysis. Our findings indicated that vancomycin TDM was associated with a lower risk of 28 day mortality (multivariable Cox proportional model, adjusted HR: 0.87; 95% CI: 0.79, 0.95; *p* = 0.002). Furthermore, after excluding 1,307 patients with positive microbiological cultures for MRSA infection, the association between vancomycin TDM and 28 day mortality remained significant (multivariable Cox proportional model, adjusted HR: 0.84; 95% CI: 0.78, 0.92; *p* < 0.001) (Table 5).

**TABLE 5:** Sensitivity analysis of the relationship between vancomycin TDM and 28 day mortality.

Sensitivity	Matching	28 day mortality,n (%)				Correlation analysis		
		Total	Non-TDM group	TDM group	P	HR	95%CI	P
Model 1 (n = 11,998)	before PSM	2,591/11,998 (21.6%)	864/5,255 (16.4%)	1727/6,743 (25.6%)	<0.001	0.87^a^	0.79∼0.95	0.002
	after PSM	1,418/6,052 (23.4%)	764/3,026 (25.2%)	654/3,026 (21.6%)	<0.001	0.90^b^	0.81∼1.00	<0.001
Model 2 (n = 16,749)	before PSM	3,318/16,749 (19.8%)	1,607/10,064 (16.0%)	1711/6,685 (25.6%)	<0.001	0.84^a^	0.78∼0.92	<0.001
	after PSM	1956/7,892 (24.8%)	1,054/3,946 (26.7%)	902/3,946 (22.9%)	<0.001	0.89^b^	0.82∼0.97	0.009

Model 1: Excluded those who ICU, stay was less than 48 h; Model 2: Excluded those who had positive microbiological cultures of MRSA.

^a^ HR, from a multivariable Cox proportional model adjusted for all covariates in Table 1.

^b^ HR, from a multivariable Cox proportional hazards model with the same strata and covariates, with additional adjustment for the propensity score.

## 4 Discussion

In this large retrospective cohort study, we found that patients in the TDM group exhibited more severe conditions and a higher 28 day mortality rate compared to those in the non-TDM group. After balancing the baseline characteristics of patients between the two groups using PSM, the mortality rate in the TDM group was lower than that in the non-TDM group. After including all covariates, the multivariate Cox model suggested that vancomycin TDM is a protective factor that can reduce the 28 day mortality rate among critically ill patients. Both the Cox proportional hazards regression analysis model and the PSM model corroborated this finding. Kaplan-Meier survival curves identified a lower 28 day mortality rate in the TDM group. Collectively, these results demonstrated that monitoring vancomycin blood concentrations was associated with a lower 28 day mortality rate in critically ill patients.

Typically, the severity of illness in critically ill patients correlates positively with both the duration of ICU admission and mortality rate (Knaus et al., 1991). In patients treated with vancomycin, increased disease severity not only prolongs hospitalization but also increases the need for TDM (Abdul-Aziz et al., 2020; Wong et al., 2014). Consequently, critically ill patients undergoing vancomycin TDM may exhibit higher mortality rates due to their more severe condition, while those not undergoing TDM, reflecting milder conditions, may present lower mortality rates. Our findings corroborate this hypothesis. If vancomycin TDM indeed mitigates the risk of mortality in critically ill patients, the severity of the underlying conditions might obscure these beneficial effects. Our analysis using multivariate Cox proportional hazards regression and PSM model robustly demonstrated that, after adjusting for covariates reflecting disease severity, monitoring vancomycin blood concentrations was associated with a significant reduction in the 28 day mortality rate in critically ill patients. Although monitoring drug concentrations alone does not constitute a complete TDM process, it is a fundamental component of TDM. It provides the basis for individualized vancomycin dosing, allowing clinicians to optimize therapy based on patient-specific PK and PD characteristics. Drug concentration monitoring enables timely dose adjustments, achieving therapeutic levels, reducing toxicity, and improving patient outcomes. Therefore, drug concentration monitoring represents an essential part of TDM and significantly reflects the application of TDM in clinical practice.

It is pertinent to note that only a limited number of studies have investigated the clinical outcomes of vancomycin dose adjustments under TDM guidance compared to those without such interventions (Ye et al., 2013; Fernández de Gatta et al., 1996; Iwamoto et al., 2003; Liu et al., 2024; Welty and Copa, 1994; Marella et al., 2020; Cardile et al., 2015). Predominantly, these investigations were derived from small-scale, single-disease studies, and non-randomized controlled trials. For instance, Machado et al. (2017) investigated the effect of antimicrobial TDM, including agents such as imipenem, meropenem, piperacillin, and vancomycin, on the prognosis of burns patients but found no evidence of prognostic improvement. A systematic review and meta-analysis investigated the advantages of vancomycin TDM and demonstrated a significant improvement in clinical efficacy (HR: 2.62; 95% CI: 1.34, 5.11; *p* = 0.005) and a marked reduction in nephrotoxicity (HR: 0.25; 95% CI: 0.13, 0.48; *p* < 0.0001), albeit without mortality data (Ye et al., 2013). In another study, Fernández de Gatta et al. (1996) found that vancomycin TDM reduced nephrotoxicity in patients with hematological malignancies; however, the sample sizes analyzed in this study were small. Another retrospective analysis of 184 MRSA infections, classified into TDM and non-TDM groups, did not report mortality outcomes (Iwamoto et al., 2003). A previous study by Huanhuan et al. compared patients with postoperative intracerebral hemorrhage who received TDM to those who did not; the analysis found no significant differences between the two groups in terms of 14 day mortality rate and the length of hospital stay (Liu et al., 2024). Welty et al. further reported that vancomycin TDM was associated with a lower incidence of nephrotoxicity, a shorter treatment duration, a reduced total dose, and a shorter hospital stay, but not mortality rates (Welty and Copa, 1994). In another study, Marella et al. compared the efficacy of vancomycin TDM in critically ill adult patients undergoing extracorporeal membrane oxygenation and found no significant difference in mortality rates between the TDM group (62.3%) and the non-TDM group (68.4%) (Marella et al., 2020). Cardile et al. found that TDM successfully achieved the initial target trough concentration of vancomycin for Gram-positive cocci infections more rapidly, particularly for MRSA infections; however, there was no significant difference between the groups in terms of treatment failure and in-hospital mortality rates (Cardile et al., 2015). To our knowledge, our present study is the first to use large-scale data to confirm that monitoring vancomycin blood concentrations can significantly reduce mortality rates in critically ill patients when compared to the administration of vancomycin without monitoring. The strength of our study lies in the provision of detailed mortality data, a large sample size, and consistent results.

Vancomycin TDM provides substantial benefits for critically ill patients in several key aspects. First, attaining therapeutic levels of vancomycin in ICUs remains a significant challenge. Previous evidence indicated that 40%–55.8% of ICU patients do not attain adequate initial trough concentrations post-administration, potentially leading to therapeutic failure (Mahmoodian et al., 2016; Obara et al., 2016; Alshehri et al., 2020; Bakke et al., 2017). The implementation of TDM for vancomycin enables clinicians to tailor pharmacotherapy more precisely, ensuring the timely attainment of therapeutic targets (Bakke et al., 2017; Truong et al., 2018; Flannery et al., 2020) and enhancing the likelihood of treatment success (Ye et al., 2013; Shahrami et al., 2016). Many studies have investigated the relationship between mortality rates and trough concentrations or AUC/MIC within a specific range (Alshehri et al., 2020; Li et al., 2022; Chen et al., 2024). If the trough concentrations or AUC/MIC ratios fall outside of these therapeutic ranges, dose adjustments can be effectively employed to align with established targets, thereby mitigating the risk of mortality (Steinmetz et al., 2015; Casapao et al., 2015; Lodise et al., 2014). Furthermore, both trough concentrations and AUC can predict potential nephrotoxicity. When high trough concentrations or AUC are detected, adjustments in dosage and administration can prevent drug-related nephrotoxicity (Hall et al., 2024; Ishigo et al., 2024), thereby reducing patient mortality (Aljefri et al., 2019; Abdelmessih et al., 2022; Li et al., 2022; Chen et al., 2024). Although TDM increases medical costs, dosage adjustment remains cost-effective for some critically ill patients (Ye et al., 2016; Fernández de Gatta et al., 1996). These considerations clearly demonstrate the necessity of implementing vancomycin TDM in critical care settings.

Our subgroup analysis showed that patients diagnosed with sepsis or septic shock had better survival outcomes from monitoring vancomycin blood concentrations compared to those without such diagnoses. Patients receiving RRT, vasopressor drugs, or mechanical ventilation showed significantly greater survival benefits from monitoring vancomycin blood concentrations compared to those who did not. Compared to patients with eGFR ≥60 mL/min/1.73 m^2^, those with eGFR <60 mL/min/1.73 m^2^ gained survival benefits from monitoring vancomycin blood concentrations, especially those with eGFR <15 mL/min/1.73 m^2^, while patients with eGFR ≥60 mL/min/1.73 m^2^ did not show survival benefits. Additionally, patients with higher severity scores (APS III ≥40, OASIS ≥30, SAPS II ≥ 30) benefited from vancomycin TDM, while those with lower severity scores did not. These findings suggest that only critically ill patients with severe conditions benefit from monitoring vancomycin blood concentrations. This indicates that monitoring vancomycin blood concentrations is especially important for critically ill patients, probably because precise dosing is necessary to balance efficacy and toxicity in severe cases. These findings are crucial for guiding clinical decisions regarding the implementation of TDM in critically ill patients receiving vancomycin.

Due to time constraints, a short ICU stay might preclude the possibility of conducting vancomycin TDM, even if it is needed. When excluding patients with an ICU stay of less than 48 h, our findings mirrored those of the entire cohort, further confirming the survival benefits of monitoring vancomycin blood concentrations for critically ill patients. Previously, most research on precise clinical guidance relating to vancomycin TDM focused on infections caused by MRSA (Steinmetz et al., 2015; Lodise et al., 2020; Bakke et al., 2017; Casapao et al., 2015; Lodise et al., 2014), whereas vancomycin was often used empirically in ICU settings (Cowley et al., 2019; Chow et al., 2020; Bostwick et al., 2019). To investigate the impact of monitoring vancomycin blood concentrations on mortality during empirical use, we excluded those with confirmed MRSA infections. Our results demonstrated clear survival benefits for severely ill patients without confirmed MRSA infection.

This study has several limitations that need to be considered. First, as a retrospective study, the non-randomized grouping of patients introduced inherent baseline disparities. To address potential selection and ascertainment biases, we used PSM to mitigate these disparities. However, residual confounding factors might have influenced prognostic outcomes for critically ill patients. Second, although our findings indicated that monitoring vancomycin blood concentrations reduced mortality in critically ill patients, our analysis did not consider cost-effectiveness. This leaves the economic feasibility of routine implementation for all critically ill patients uncertain. Third, monitoring vancomycin blood concentrations is not equivalent to completing the full TDM process; it merely represents a foundational step. Furthermore, due to the limitations of our data sources, we were unable to determine which patients underwent AUC monitoring and which only had trough levels measured. Moreover, it is unclear who led the TDM implementation, who interpreted the results, and how the treatment regimen was adjusted based on the monitoring results. Additionally, blood concentration monitoring may serve as a surrogate marker for overall higher quality of care, and the influence of other unknown variables on patient outcomes cannot be excluded. Fourth, due to the complex clinical scenarios of critically ill patients and the prevalence of infections, a definitive causal relationship between monitoring vancomycin blood concentrations and patient outcomes in critical care settings has yet to be established. Therefore, our findings should be interpreted with caution.

## 5 Conclusion

This cohort study showed that monitoring vancomycin blood concentrations is associated with a significantly lower 28 day mortality rate in critically ill patients, with greater survival benefits observed in those with more severe conditions, underscoring the importance of routine vancomycin TDM in these patients.

## Data Availability

The original contributions presented in the study are included in the article/Supplementary Material, further inquiries can be directed to the corresponding authors.
