# Supplementary material for: Monitoring vancomycin blood concentrations reduces mortality risk in critically ill patients: a retrospective cohort study using the MIMIC-IV database
Source: Front Pharmacol. 2024 Nov 14;15:1458600. doi: 10.3389/fphar.2024.1458600 (PMC11602295; doi:10.3389/fphar.2024.1458600)
Supplement: Supplementary file 1 [file Table1.DOCX]

Supplementary Material

# Supplementary Table 1：detailed information on missing data of variables in the study

| variable | inventory | N missing | missing rate |
| --- | --- | --- | --- |
| Heart rate | 18011 | 45 | 0.25% |
| MAP | 18009 | 47 | 0.26% |
| Respiratory rate | 18005 | 51 | 0.28% |
| Temperature | 17133 | 923 | 5.11% |
| Spo2 | 18004 | 52 | 0.29% |
| WBC | 18002 | 54 | 0.30% |
| Hemoglobin | 18001 | 55 | 0.30% |
| Hematocrit | 18007 | 49 | 0.27% |
| Platelets | 18003 | 53 | 0.29% |
| Creatinine | 18018 | 38 | 0.21% |
| BUN | 18012 | 44 | 0.24% |
| Glucose | 17942 | 114 | 0.63% |
| Potassium | 17995 | 61 | 0.34% |
| Bicarbonate | 18011 | 45 | 0.25% |
| SOFA score | 18012 | 44 | 0.24% |
| Vancomycin dose | 17500 | 556 | 3.08% |
| Vancomycin time | 17465 | 591 | 3.27% |

The other variables in this study do not missing.
