# Supplementary figures and images for: Monitoring vancomycin blood concentrations reduces mortality risk in critically ill patients: a retrospective cohort study using the MIMIC-IV database
Source: Front Pharmacol. 2024 Nov 14;15:1458600. doi: 10.3389/fphar.2024.1458600 (PMC11602295; doi:10.3389/fphar.2024.1458600)

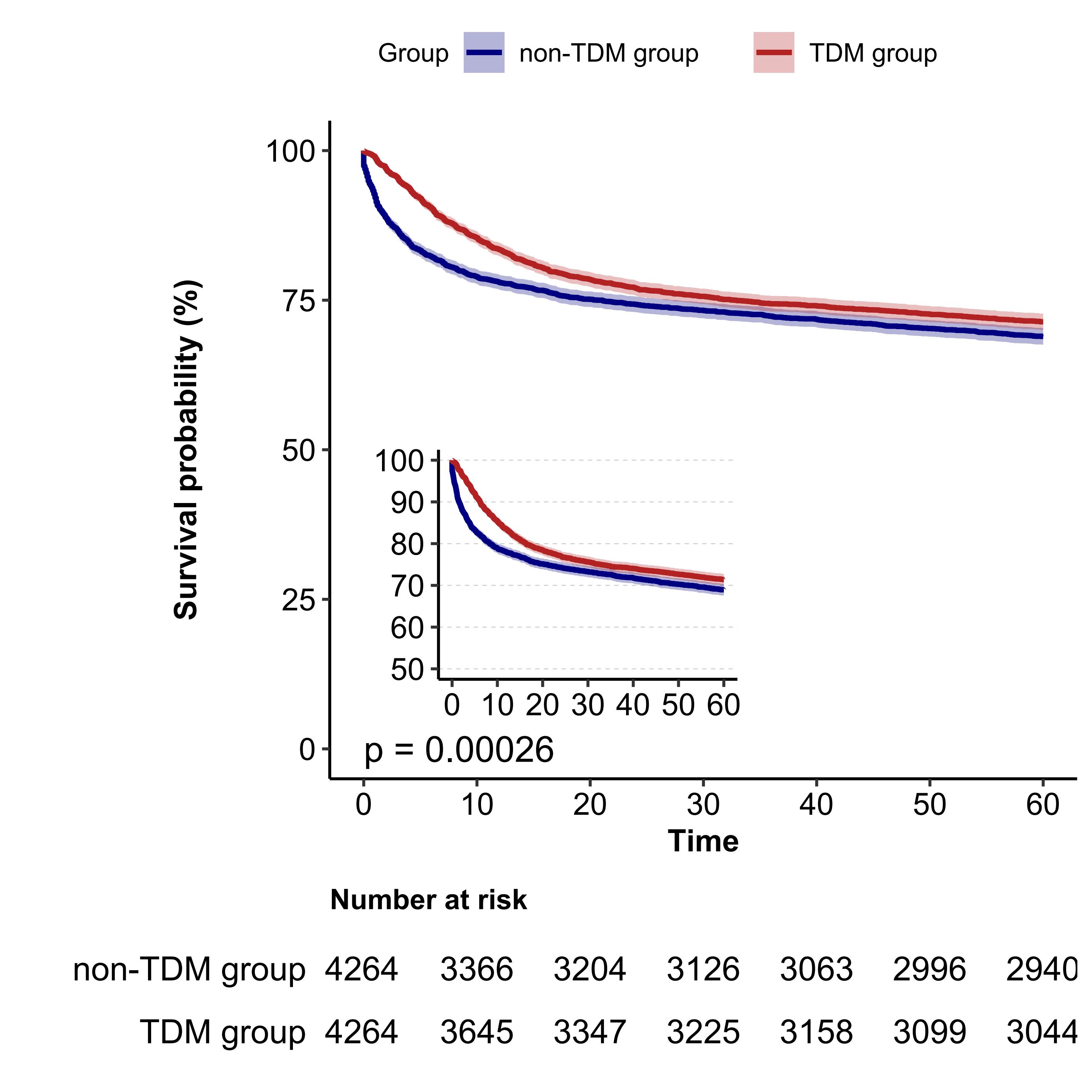

Supplement: Supplementary file 2 [file Image1.JPEG]

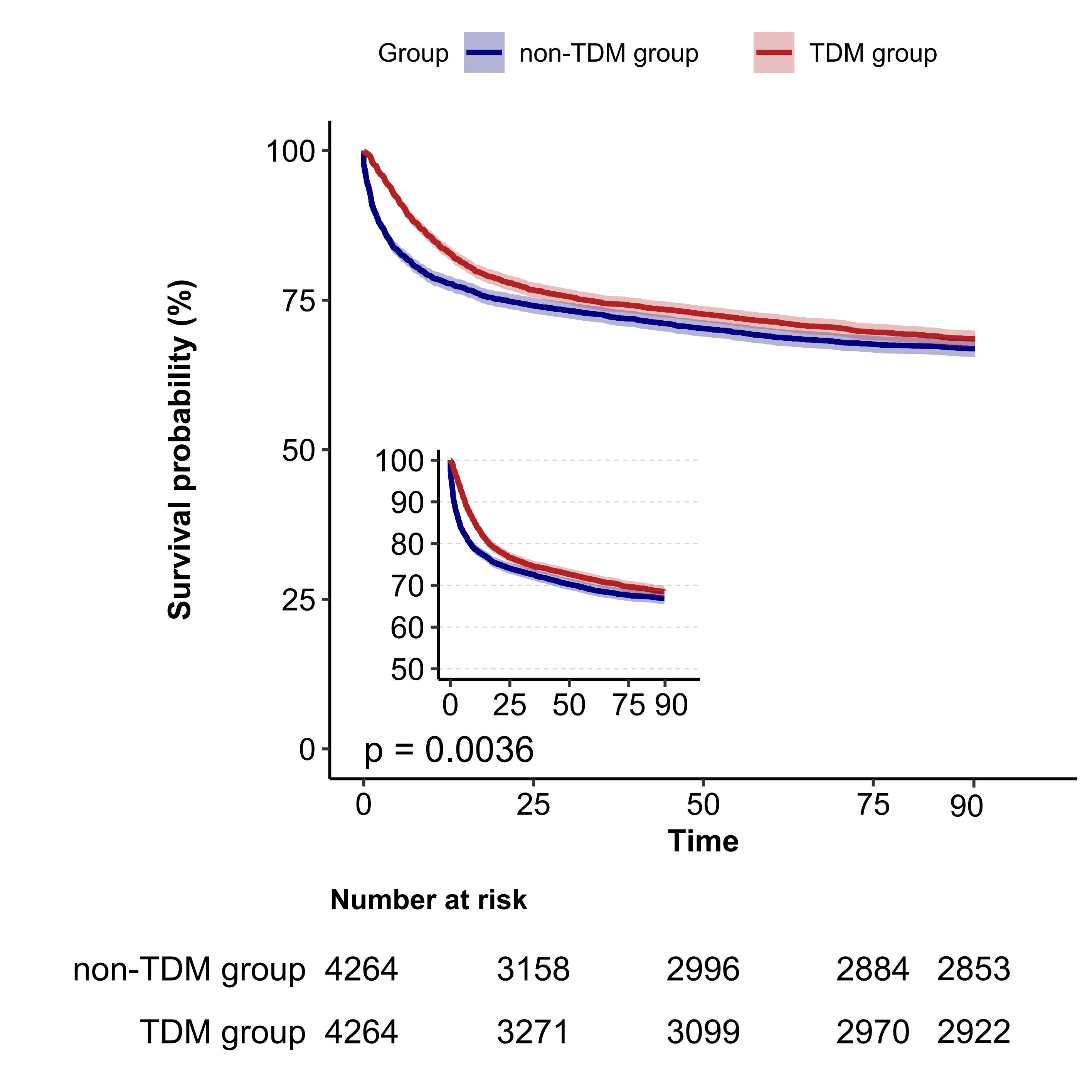

Supplement: Supplementary file 3 [file Image2.JPEG]
